# Construction of a prognostic model based on ferroptosis- and mitochondrial metabolism-related genes for patients with breast cancer

**DOI:** 10.1097/MD.0000000000043307

**Published:** 2025-07-18

**Authors:** Xue Han, Wurina Chen

**Affiliations:** a Medical Oncology, Hulunbuir People’s Hospital, Hulunbuir, Inner Mongolia, China.

**Keywords:** breast cancer, ferroptosis, metabolism, mitochondrion, prognosis

## Abstract

Mitochondrial metabolism (MM)-mediated ferroptosis plays a critical role in breast cancer (BC). However, the potential targets based on ferroptosis and MM in BC remain poorly understood. This study aimed to explore the prognostic role of ferroptosis- and MM-related genes (FPMMs) in BC. Differentially expressed FPMMs were identified, and functional analyses were performed. Univariate Cox, LASSO, and multivariate Cox regression analyses were used to screen hub genes, and a prognostic risk model was then constructed and validated in external datasets. Gene set variation analysis was conducted to investigate their regulatory functions. Furthermore, immune infiltration analysis was performed using the “quantiseq” algorithm. We identified 206 differentially expressed FPMMs. A prognostic risk model consisting of 6 genes (BRD4, FLT3, SIAH2, CS, EMC2, and PI3KCA) was constructed, exhibiting good predictive capability and stability. These 6 prognostic genes were dysregulated in BC, with PI3KCA exhibiting the highest mutation frequency. Gene set variation analysis further revealed that the PI3K-AKT-mTOR signaling was suppressed in BC. In addition, the risk score based on the prognostic model was associated with immune infiltration, particularly with B cells, T cells, CD4, and dendritic cells. Our study highlights the potential of the prognostic model based on FPMMs as a valuable tool for BC prognosis prediction.

## 1. Introduction

Breast cancer (BC) is the most commonly diagnosed cancer among women worldwide, leading to 15.5% female deaths.^[1,2]^ Although advancements in diagnosis and treatment strategies have occurred, the prognosis for BC patients remains variable, with factors such as tumor subtype, stage, and molecular characteristics influencing outcomes.^[3]^ Clinical, pathological, and molecular characteristics are commonly employed for predicting treatment response and clinical prognosis.^[4]^ However, traditional clinical and pathological features such as tumor size, lymph node status, and histological grade often fail to fully capture the biological complexity of tumors and are insufficient to accurately predict the prognosis of BC patients.^[5]^ Although certain molecular markers have shown some value in the monitoring of BC, their specificity and sensitivity remain limited.^[6]^ Therefore, it is urgent to identify more sensitive and specific biomarkers to improve risk stratification, guide personalized treatment, and more accurately predict patient prognosis.

Ferroptosis is a distinctive iron-dependent non-apoptotic form of cell death characterized by intracellular iron overload and lethal lipid peroxidation accumulation.^[4]^ The biochemical process of ferroptosis mainly involves the depletion of intracellular glutathione (GSH) and decreased activity of glutathione peroxidase 4 (GPX4).^[7]^ The small molecule erastin initiates ferroptosis by suppressing the function of cystine-glutamate antiporter (SLC7A11), resulting in the reduction of cellular cysteine and GSH levels.^[8]^ Excessive lipid peroxides that are not fully metabolized via GPX4-catalyzed reduction reactions result in the accumulation of reactive oxygen species (ROS).^[9]^ Morphological features of ferroptosis include mitochondrial shrinkage, reduced or loss of mitochondrial cristae, and increased mitochondrial membrane density.^[10]^ Mitochondria, serving as central hubs for energy metabolism, including fatty acid oxidation, the tricarboxylic acid cycle, and the electron transport chain, play crucial roles in promoting ferroptosis through metabolic pathways.^[11]^ During erastin-induced ferroptosis, reduced glycolytic flux is observed in cells with disrupted glycolysis, while mitochondrial oxidative phosphorylation is enhanced, ultimately leading to cell ferroptosis.^[12]^

Emerging evidence suggests that ferroptosis and mitochondrial metabolism (MM) play crucial roles in BC progression and treatment response.^[13,14]^ Given the intricate interplay between ferroptosis and MM, there is growing interest in exploring their potential as therapeutic in BC. MTHFD2, a mitochondrial bifunctional enzyme in glucose metabolism, has been identified as a ferroptosis regulator in triple-negative BC.^[15]^ Epothilone B, used to treat metastatic BC in clinical, promotes mitochondrial ROS to induce ferroptosis.^[16]^ However, the targets and underlying mechanisms based on ferroptosis and MM in treating BC need more exploration.

In this study, we aimed to develop a comprehensive prognostic model utilizing a panel of ferroptosis- and MM-related genes (FPMMs) to predict outcomes for patients with BC. By leveraging public genomic datasets and bioinformatics tools, we seek to identify gene signatures associated with disease overall survival (OS). Through the development of this prognostic model, we endeavor to enhance our understanding of the molecular mechanisms underlying BC progression and uncover novel therapeutic targets.

## 2. Methods

### 2.1. Data resource

The clinical data of patients with BC were downloaded from the TCGA database (https://portal.gdc.cancer.gov). The gene expression profile GSE20685 for BC patients was obtained from the GEO database (http://www.ncbi.nlm.nih.gov/geo). The ferroptosis-related genes were screened from the FerrDb database (http://www.zhounan.org/ferrdb). MSigDB database (https://www.gsea-msigdb.org/gsea/msigdb) was searched to acquire the MM-related genes.

### 2.2. Identification of differentially expressed FPMMs in BC

Differentially expressed genes (DEGs) between normal and tumor samples were identified using the R package “Limma” based on the TCGA dataset, with criteria of *P* value < 0.05 and |log_2_fold change| ≥ log_2_(1.5). A volcano plot was used to visualize the results. The differentially expressed FPMMS were screened by intersecting the DEGs, ferroptosis-related genes, and MM-related genes through a Venn diagram using the R package “VennDiagram.”

### 2.3. Enrichment analyses for the FPMMs

Using the R package “clusterProfiler,” Gene ontology (including biological process, cellular component, and molecular function) and Kyoto Encyclopedia of Genes and Genomes enrichment analyses were conducted.

### 2.4. Construction of prognostic risk model

Univariate Cox regression was performed on the FPMMs to screen prognosis-related genes, with OS as the dependent variable. LASSO regression was conducted on these identified genes to mitigate overfitting using the “glmnet” package in R. Finally, hub prognostic genes were identified through multivariate Cox regression and used to construct a prognostic model for BC. The risk scores of each patient in TCGA were calculated as:


$$\begin{array}{l} risk\;score\;=\;\underset{i=1}{\overset{n}{\sum }}\;exp_{RNAi}*Coef_{RNAi} \end{array}$$

Where exp_RNAi_ means the expression levels of hub genes, and Coef_RNAi_ represents the regression coefficients of hub genes from the multivariate Cox regression analysis.

### 2.5. Validation of prognostic risk model

BC patients in the TCGA were divided into low- and high-risk groups based on the median value of the risk scores. Differences in OS between different risk groups were visualized through Kaplan–Meier curves. The predictions of the risk model were further assessed through a receiver operating characteristic (ROC) curve using the R package “timeROC.” Finally, the stability of this model was validated in the GSE20685 dataset.

### 2.6. Nomogram construction

The association of risk score with clinical parameters, including gender, age, pathology stage, T, N, and M stages, was investigated. The nomogram was constructed based on risk scores and these clinical parameters, and a calibration curve was used to determine the 45-degree dashed lines, which means the best prediction of the nomogram. The R packages “rms,” “regplot,” and “survival” were employed.

### 2.7. Gene set variation analysis (GSVA) of prognostic genes

Based on the prognostic gene expression median level, the TCGA samples were divided into high and low expression groups. GSVA enrichment analysis was executed using signaling pathway datasets from the MSigDB database.

### 2.8. Immune infiltration and mutation analysis

This study determined the infiltration of immune cells between low- and high-risk groups using the “quantiseq” algorithm in the IOBR package, and those showing significant differences were visualized using ggplot. Additionally, the correlation of immune cells identified using “quantiseq” with prognostic genes and risk score was analyzed through the Pearson method.

For the mutation analysis, the mutation quality and frequency of 6 prognostic genes in 2 risk groups were analyzed through the R package “Maftools.”

### 2.9. Statistical analysis

Statistical analyses were conducted using R software version 4.1.2 (https://www.R-project.org/). The FPMM-related prognostic model was established through univariate LASSO and multivariate Cox regression. The Kaplan–Meier method was employed to compare the OS between 2 different risk groups. Evaluation of the model was performed using the “timeROC” package in R, which generated the ROC curve and the corresponding area under the ROC curve (AUC) for 3-, 5-, and 10-year survival. Correlation analysis was performed using the Pearson method. *P* < .05 was considered statistically significant.

## 3. Results

### 3.1. Functional enrichment analysis of differentially expressed FPMMs in BC

**Figure 1.**
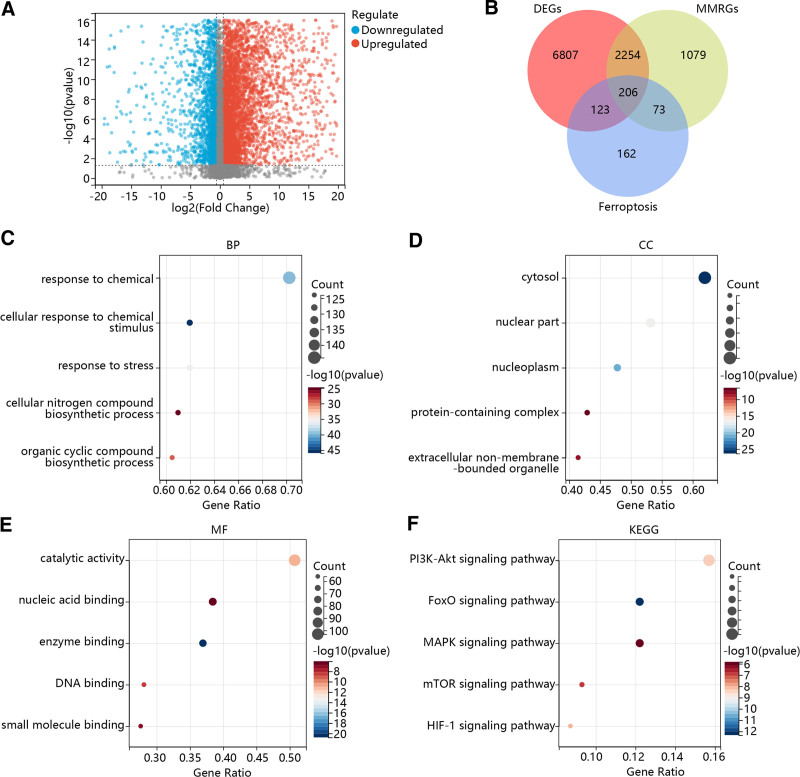
Functional enrichment analysis of differentially expressed FPMMs in BC. (A) Volcano plot of DEGs in BC. Red dots are upregulated genes, and blue dots are downregulated genes. (B) The Venn diagram of DEGs, MM-related genes, and ferroptosis-related genes. (C–E) GO enrichment analysis. (F) KEGG pathway enrichment analysis. BC = breast cancer, BP = biological process, CC = cellular component, DEGs = differentially expressed genes, FPMMs = ferroptosis- and mitochondrial metabolism-related genes, GO = Gene Ontology, KEGG = Kyoto Encyclopedia of Genes and Genomes, MF = molecular function, MM = mitochondrial metabolism.

Firstly, 1118 tumor tissue samples and 113 normal tissue samples from the TCGA cohort were included, and 9390 DEGs between them were identified. There were 5718 upregulated and 3672 downregulated genes in the tumor tissues compared to the normal tissues (Fig. 1A). Then, 3612 MM-related genes and 564 ferroptosis-related genes were screened from the MSigDB and FerrDb databases, respectively. After intersecting the DEGs, MM-related genes, and ferroptosis-related genes, 206 differentially expressed FPMMs were acquired (Fig. 1B). Further enrichment analyses were performed on these FPMMs to explore their biological functions and associated pathways. The top 5 biological processes were response to chemical, cellular response to chemical stimulus, response to stress, cellular nitrogen compound biosynthetic process, and organic cyclic compound biosynthetic process (Fig. 1C). The cellular components were correlated with cytosol, nuclear part, nucleoplasm, protein-containing complex, and extracellular non-membrane-bounded organelle (Fig. 1D). As for molecular function, these genes were enriched in the catalytic activity, nucleic acid binding, enzyme binding, DNA binding, and small molecule binding (Fig. 1E). In addition, the Kyoto Encyclopedia of Genes and Genomes analysis revealed that these FPMMs were involved in the PI3K-Akt signaling pathway, FoxO signaling pathway, MAPK signaling pathway, mTOR signaling pathway, and HIF-1 signaling pathway (Fig. 1F).

### 3.2. Construction of FPMM-based prognostic risk model

**Figure 2.**
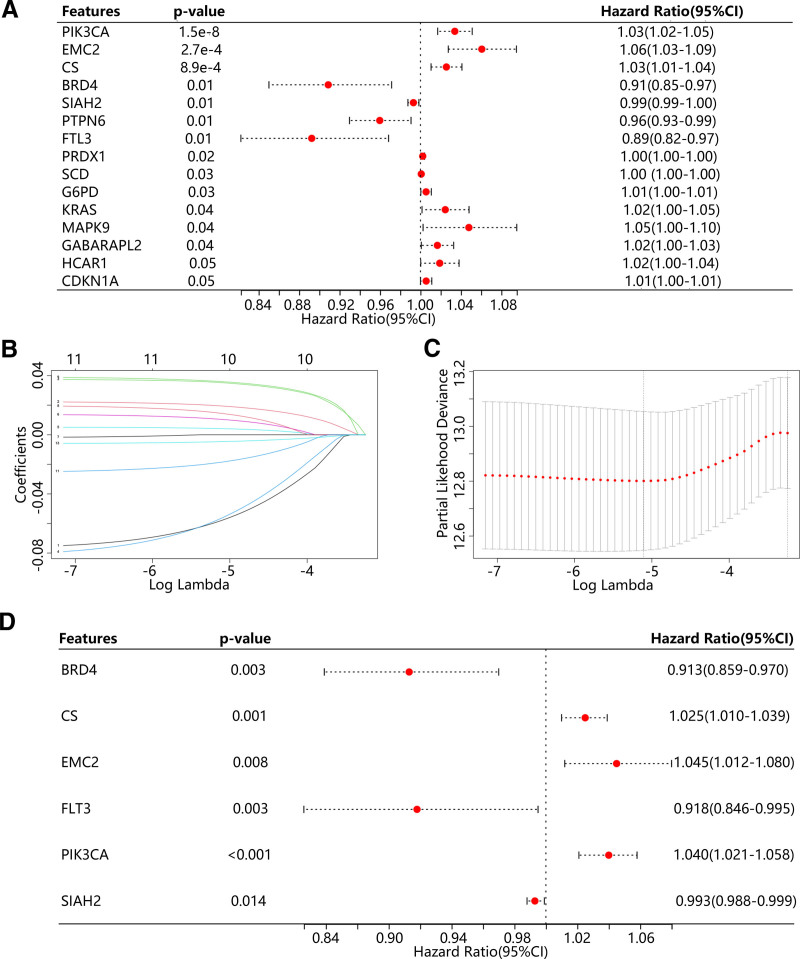
Construction of FPMM-based prognostic risk model. (A) Univariate Cox regression analysis based on the differentially expressed FPMMs. (B–C) LASSO regression analysis removed redundant features. (D) Multivariate Cox regression analysis identified 6 FPMM-related prognostic genes. FPMMs = ferroptosis- and mitochondrial metabolism-related genes.

Using the univariate Cox regression analysis, we identified 15 FPMM-related prognostic genes (Fig. 2A). The LASSO regression model was screened using *λ* = 0.03894656, and its cross-validation results were visualized in Figure 2B and C. Ten FPMM-related prognostic genes identified by LASSO were then seeded into the multivariate Cox regression analysis. Finally, 6 genes were identified by the multivariable Cox regression analysis and used to construct a prognostic risk model. Among the 6 genes, BRD4, FLT3, and SIAH2 were identified as protective features, while CS, EMC2, and PI3KCA were considered detrimental (Fig. 2D).

### 3.3. Assessment of the FPMM-based prognostic risk model

**Figure 3.**
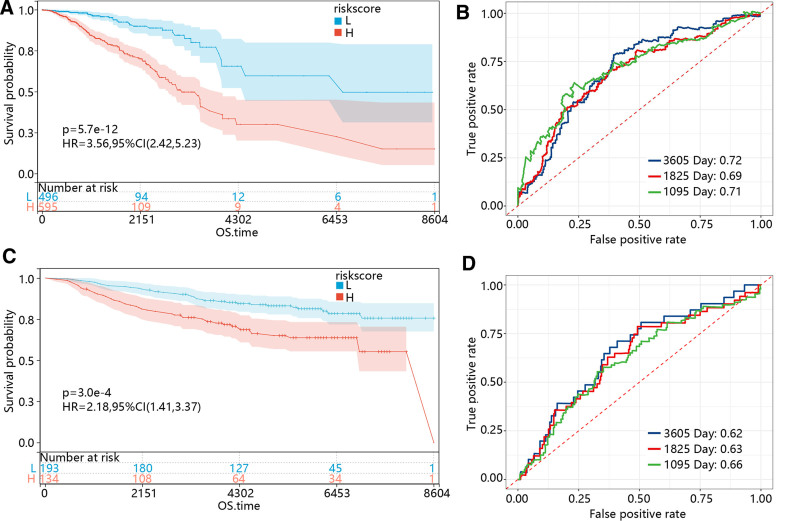
Assessment of the FPMM-based prognostic risk model. (A) Kaplan–Meier curve of BC patients in the low-risk and high-risk groups based on the TCGA cohort. (B) ROC curve at 3-, 5-, and 10-year for BC patients in the TCGA cohort. (C) Kaplan–Meier curve of BC patients in the low-risk and high-risk groups based on the GSE20685 dataset. (D) ROC curve at 3-, 5-, and 10-year for BC patients in the GSE20685 dataset. BC = breast cancer, FPMMs = ferroptosis- and mitochondrial metabolism-related genes, ROC = receiver operator characteristic.

To assess the prognosis value of the model, patients in the TCGA cohort were divided into high-risk and low-risk groups based on the median value of risk scores in each sample. The risk score was calculated as “risk score = −0.091*BRD4 + 0.024*CS + 0.044*EMC2 + −0.086*FLT3 + 0.039*PIK3CA + −0.007*SIAH2.” Kaplan–Meier curve revealed that patients with low-risk scores had longer survival time than patients with high-risk scores (Fig. 3A). The AUC values of 3-, 5-, and 10-year survival were 0.71, 0.69, and 0.72, respectively, indicating a good predictive performance (Fig. 3B). Furthermore, an external dataset of GSE20685 was used to further validate the stable performance of this model. As shown in Figure 3C and D, a poor prognosis was observed in the high-risk group, and the AUC values of 3-, 5-, and 10-year survival were 0.66, 0.63, and 0.62. These results indicate that this prognostic model exhibits a preferable predictive capability and stability.

### 3.4. Independence of the FPMM-based risk score from clinical parameters of BC

**Figure 4.**
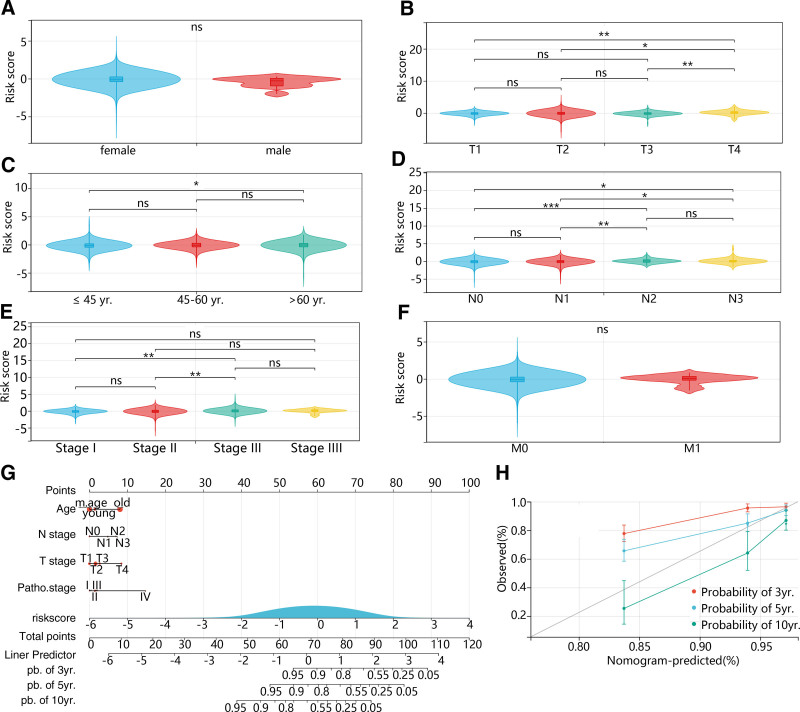
Independence of the FPMM-based risk score from clinical parameters of BC. (A–F) Risk score differences in gender, age, pathology, T, N, and M stages. (G) Nomogram of clinical parameters. (H) Calibration curves for 3-, 5-, and 10-year.*P < .05, **P < .01, ***P < .001. BC = breast cancer, FPMMs = ferroptosis- and mitochondrial metabolism-related genes, ns = no significance, patho. = pathology, pb. = probability, yr. = year.

The association of prognosis with BC clinical factors was evaluated. We observed that the risk score showed no significant difference between different genders and M stages (Fig. 4A and F). However, age, T, N, and pathology stages significantly affected BC prognosis, with different statuses showing different risk scores (Fig. 4B–E). The nomogram showed that the risk model had high accuracy for diagnosing BC patients survival at 3-year, 5-year, and 10-year (Fig. 4F). The age, T, N, and pathology stages significantly contribute to the model, with the pathology stage being the most among these factors (Fig. 4F). Furthermore, the calibration curve revealed that the model had a good prediction for 3-, 5-, and 10-year survival time, and the prediction at 5-year was the best (Fig. 4G).

### 3.5. Expression levels of FPMM-related prognostic genes and mutation analysis

**Figure 5.**
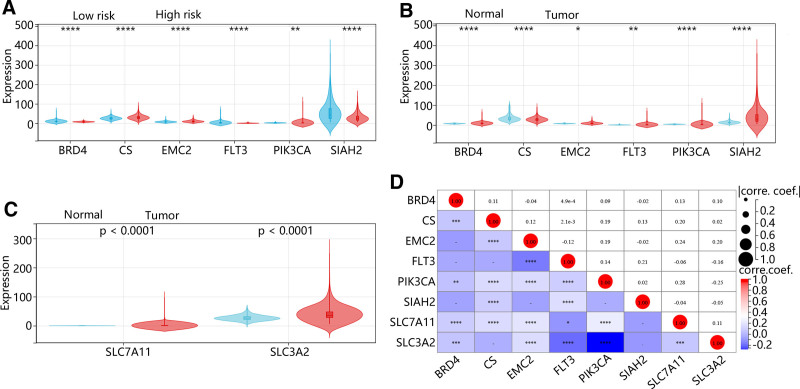
Expression levels of FPMM-related prognostic genes. (A) Expression levels of 6 prognostic genes in low- and high-risk groups. (B) Expression levels of 6 prognostic genes in normal and tumor samples. (C) Expression levels of SLC7A11 and SLC3A2 in normal and tumor samples. (D) Pearson correlation of 6 prognostic genes with SLC7A11 and SLC3A2. *P < .05, **P < .01, ***P < .001, ****P < .0001. coef. = coefficient, corre. = correlation, FPMMs = ferroptosis- and mitochondrial metabolism-related genes.

**Figure 6.**
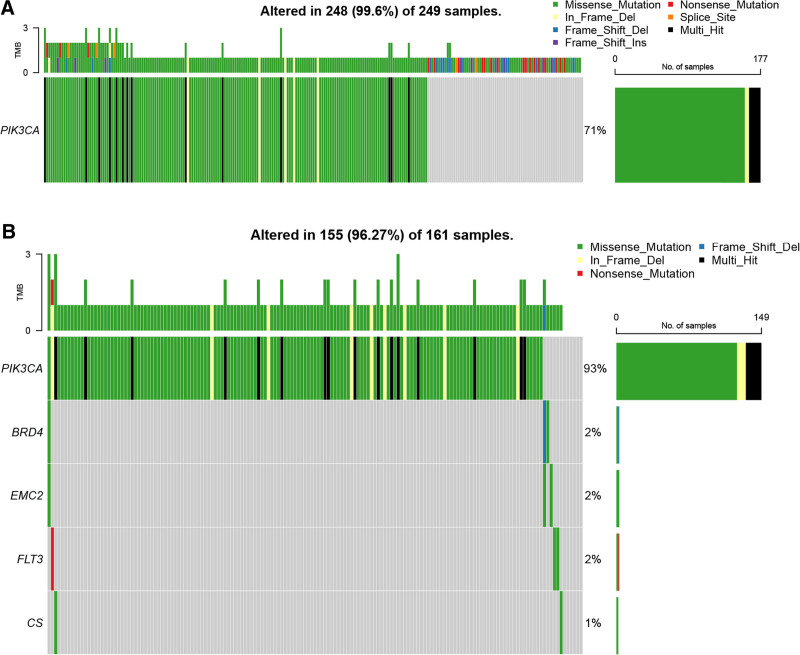
Mutation analysis. (A) Mutation frequency of prognostic genes in the low-risk group. (B) Mutation frequency of prognostic genes in the high-risk group.

The expression of 6 prognostic genes was validated in the TCGA dataset. The expression levels of protective factors BRD4, FLT3, and SIAH2 were lower whereas detrimental factors CS, EMC2, and PIK3CA levels were higher in the high-risk group than those in the low-risk group (*P* < .01, Fig. 5A). When comparing the tumor tissues and the normal tissues, the expression of BRD4, EMC2, FLT3, and SIAH2 was upregulated in the tumor tissues. In contrast, CS and PIK3CA expression were reduced in the tumor tissues (*P* < .01, Fig. 5B). Mechanisms of MM-modulated ferroptosis in inducing the malignancy of BC were further explored. SLC7A11 and SLC3A2 are the compounds of antiporter System xc, which play a crucial role in ferroptosis.^[17]^ Our data revealed that the expression of SLC7A11 and SLC3A2 was upregulated in the tumor tissues compared to the normal tissues (*P* < .0001, Fig. 5C). Further Pearson correlation analysis showed that SLC7A11 was significantly related to BRD4, CS, EMC2, and PIK3CA, with a negative association with FTL3 (*P* < .05, Fig. 5D). Besides, SLC3A2 was positively correlated with BRD4 and EMC2 while negatively related to FLT3 and PIK3CA (*P* < .001, Fig. 5D). Furthermore, mutation analysis revealed that PIK3CA was the gene mutating in both risk groups with the highest mutation frequency (93% in the high-risk group), and the other 5 genes only mutated in the high-risk group (Fig. 6A and B).

### 3.6. GSVA analysis of prognostic genes

**Figure 7.**
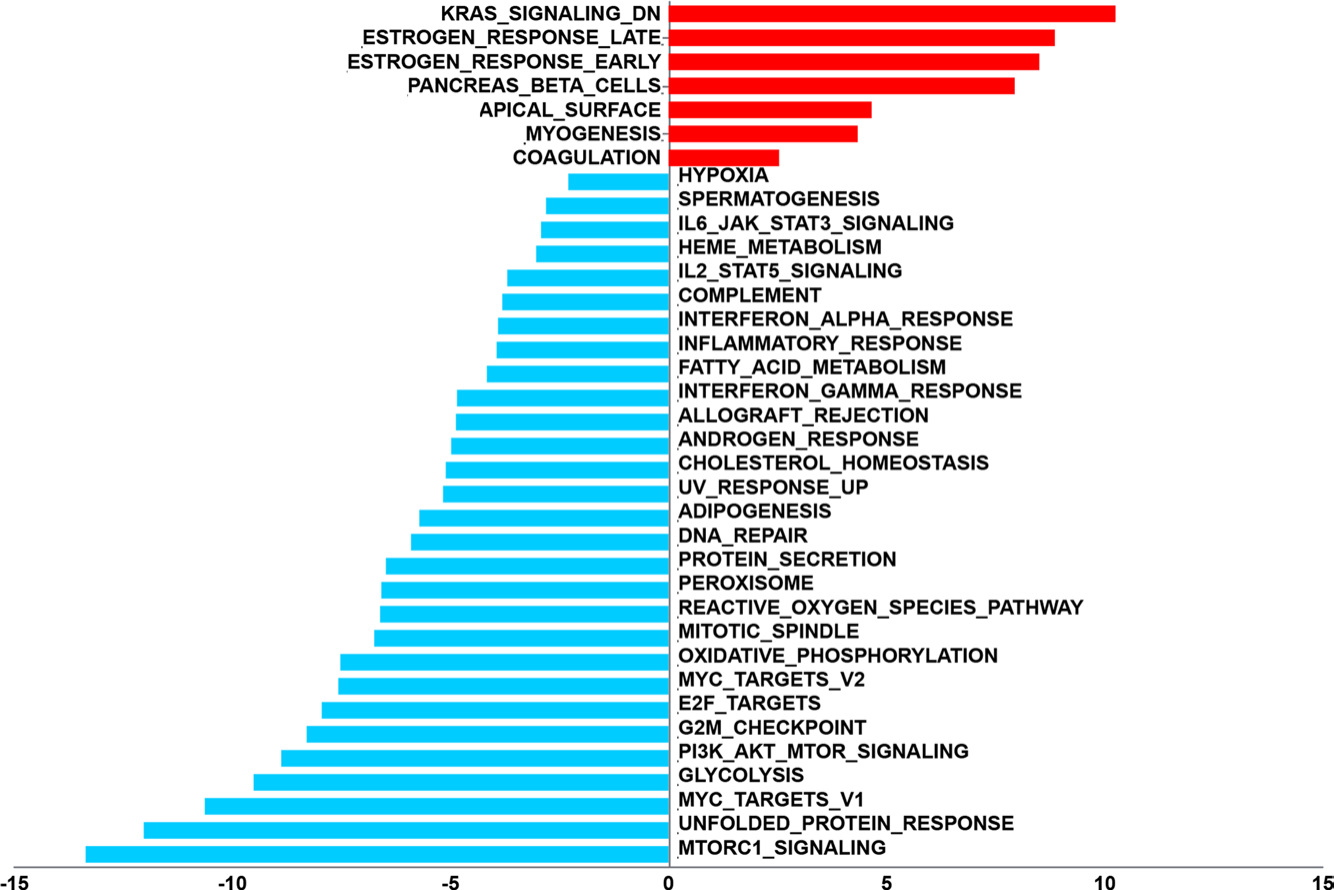
GSVA analysis of prognostic genes. GSVA = gene set variation analysis.

To deepen our exploration into the regulatory functions of prognostic genes in disease, we employed GSVA to analyze the pathways activated by these genes. The results demonstrated that KRAS signaling, estrogen response, pancreas beta cells, apical surface, and myogenesis were activated (Fig. 7). The mTORC1 signaling, unfolded protein response, Myc targets v1, glycolysis, and PI3K-AKT-mTOR signaling were inhibited (Fig. 7). All of these pathways were tightly related to tumorigenesis.

### 3.7. Immune infiltration analysis

**Figure 8.**
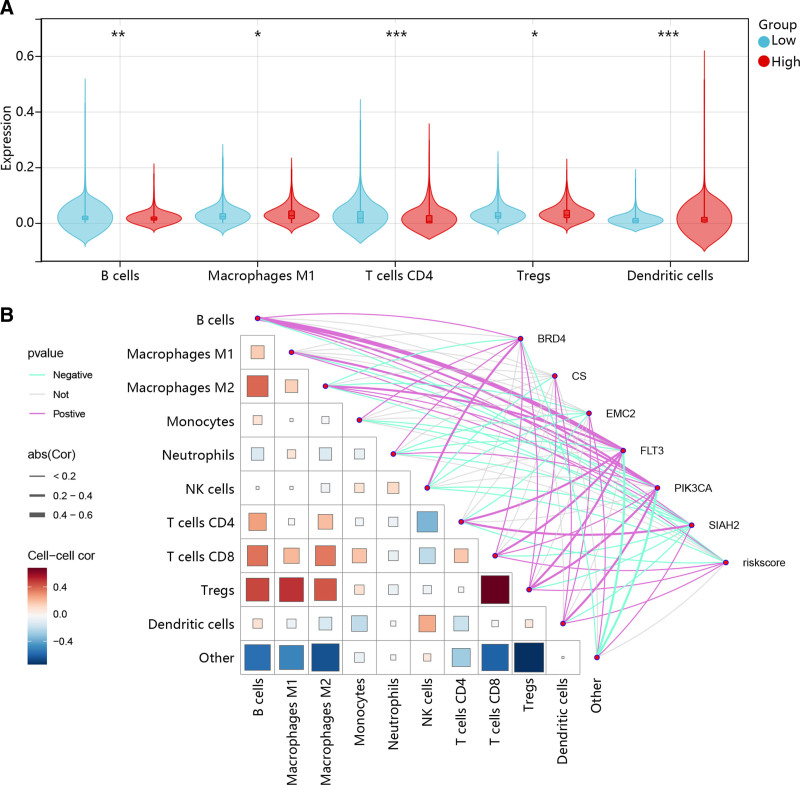
Immune infiltration analysis. (A) Immune cell infiltration in high- and low-risk groups using “quantiseq” algorithm. (B) Correlation between immune cells and correlation of immune cells with 6 prognostic genes or risk scores. *P < .05, **P < .01, ***P < .001.

Considering the importance of the immune microenvironment in BC progression, we explored the immune infiltration using the “quantiseq” algorithm. Five immune cells (B cells, macrophages M1, T cells CD4, Tregs, and dendritic cells [DCs]) showed significant infiltration differences between different risk groups, with higher levels of macrophages M1, Tregs, and DCs in the high-risk group (Fig. 8A). Additionally, as shown in Figure 8B, most of these immune cells were associated with risk scores, and 6 prognostic genes were significantly correlated with these immune cells.

## 4. Discussion

BC remains a significant challenge to the health of women worldwide.^[4]^ As a highly heterogeneous solid tumor, BC’s prognosis is poor.^[18]^ Therefore, identifying novel prognostic markers and more accurate prognostic models is crucial for improving the treatment of BC patients. Ferroptosis, a regulated necrotic process driven by iron-dependent lipid peroxidation, plays a critical role in suppressing tumorigenesis.^[19]^ The occurrence of ferroptosis is closely associated with mitochondria, and ferroptosis is regulated by MM, with alterations in MM occurring during the process of ferroptosis. Previous studies have explored the relevance of ferroptosis or MM-related genes to BC.^[20,21]^ However, limited research investigates the combined therapeutic efficacy of these 2 mechanisms in BC.

Ferroptosis is a consequence of an imbalance between oxidation and antioxidants within cells.^[22]^ Intracellular free iron catalyzes ROS through the Fenton reaction, leading to the accumulation of lipid peroxides and ultimately triggering ferroptosis. Ferroptosis inducers promote mitochondrial ROS production, inducing mitochondrial permeability transition pore opening, mitochondrial collapse, and ATP depletion.^[23]^ Cysteine plays a role in GSH synthesis. In cells deficient in cysteine, MM is significantly enhanced, promoting GSH depletion, ROS production, and ferroptosis.^[24]^ Mitochondrial ROS functions as a positive feedback in mitochondrial homeostasis and ferroptosis processes. Impairment of CDGSH iron-sulfur domain 2 function in BC induces disruption of the mitochondrial labile iron pool, leading to increased mitochondrial ROS levels and activation of ferroptosis.^[25]^ Targeting MM-modulated ferroptosis provides a novel insight for BC treatment.

Through univariate LASSO and multivariate Cox regression analysis, the present study constructed a prognostic model with 6 FPMMs, including BRD4, FLT3, SIAH2, CS, EMC2, and PI3KCA. The model exhibited good performance in prediction and stability. All of these 6 genes have been previously identified as ferroptosis-related genes, among which BRD4 and FLT3 were found to be biomarkers in BC.^[14,26]^ Our data revealed that BRD4, FLT3, and SIAH2 were the protective factors while CS, EMC2, and PIK3CA were the detrimental factors for BC patients. We found that, except for SIAH2, the other 5 genes were significantly correlated with SLC7A11 and SLC3A2. SLC7A11 and SLC3A2, as components of the system Xc-, play crucial roles in regulating intracellular levels of GSH and cellular response to oxidative stress, which are essential for regulating ferroptosis.^[27]^ Ferroptosis is induced by BRD4 downregulation.^[28]^ Inhibition of BRD4 enhances cell cycle arrest and ROS accumulation, ultimately promoting ferroptosis in BC.^[29]^ FLT3 inhibitors suppress the generation of ROS and lipid peroxidation.^[30]^ SIAH2 is a regulator of ROS; its expression downregulation was reported to elevate the protein expression of HO-1.^[31]^ HO-1 is the main source of intracellular iron, and its overactivation accelerates cellular ferroptosis.^[32]^ In BC cells, upregulation of HO-1 inhibits cancer cell proliferation and invasion.^[33]^ CS is an enzyme in mitochondria involved in the tricarboxylic acid and exists in almost all cells that have oxidative metabolism, which is related to ferroptosis.^[34,35]^ EMC2 is sensitive to erastin, and its overexpression correlates with poor prognosis in BC.^[36]^ PIK3CA is an oncogenic gene. Previous research revealed that PIK3CA inactivation sensitized cancer cells to ferroptosis.^[37]^ Additionally, PIK3CA mutation has been reported to be related to BC prognosis.^[38]^ Based on TCGA data, we found that in the different risk groups determined by the risk scores, PIK3CA had the highest mutation frequency among the 6 prognostic genes.

In our study, BRD4, FLT3, and SIAH2 were expressed at lower levels in the high-risk group, while CS and PIK3CA were highly expressed in the high-risk group. Interestingly, we observed an opposite expression pattern for these genes in tumor tissues. Specifically, BRD4, FLT3, and SIAH2 were upregulated in tumor tissues, whereas CS and PIK3CA were downregulated. This seemingly contradictory finding may be attributed to multiple factors. First, it is important to note that the stratification into high- and low-risk groups was based on intrinsic features among tumor patients rather than comparing tumor and normal tissues. Therefore, the model reflects the impact of intratumoral heterogeneity on prognosis rather than the overall gene expression changes during tumorigenesis. Previous studies have shown that BRD4 has distinct genetic subtypes with different functional states,^[39]^ and high expression of certain subtypes may be associated with a favorable prognosis. Similarly, although FLT3 is upregulated in BC,^[40]^ Chen et al reported that its expression is positively correlated with chemokines of NK cells, DCs, and T cells, suggesting that high FLT3 expression may enhance anti-tumor immunity and thus contribute to better prognosis. Moreover, bulk RNA-seq data represent the average gene expression of all cell types within a sample, which may obscure gene-specific expression patterns in critical tumor subpopulations. For instance, prior studies have demonstrated that PIK3CA expression is elevated in basal-like, HER2-positive, and triple-negative non-basal (TNnon-B) BC subtypes, while significantly reduced in Luminal subtypes.^[41]^ Given that our data are based on bulk RNA-seq, the overall averaged expression may underestimate PIK3CA’s expression in high-risk subtypes, while the model still captures its upregulation in these subgroups and associates it with poor prognosis. Collectively, our findings reflect the complex relationships among gene mutation status, expression regulation, and tumor heterogeneity. This highlights the necessity of integrating multi-dimensional data, including gene subtypes, immune microenvironment interactions, and molecular classification of tumors, to comprehensively understand the roles of specific genes in cancer.

Further GSVA analysis revealed the pathway states associated with the 6 prognostic genes. The results revealed some activated pathways, such as KRAS signaling, estrogen response, pancreas beta cells, apical surface, and myogenesis, as well as suppressed pathways including mTORC1 signaling, unfolded protein response, Myc targets v1, glycolysis, and PI3K-AKT-mTOR signaling. Estrogen signaling plays a significant role in the BC microenvironment. High estrogen-responsive BC is associated with Myc targets, metabolic signaling pathways, and mTORC1 signaling transduction gene sets, while low estrogen responsiveness is associated with KRAS signaling transduction.^[42]^ KRAS signaling refers to the intracellular signaling pathways activated by the KRAS protein, a member of the RAS family of small GTPases. KRAS signaling plays a crucial role in regulating various cellular processes, including cell proliferation, survival, differentiation, and migration.^[43]^ In BC, aberrant activation of KRAS signaling pathways can promote tumor growth, metastasis, and resistance to therapy. The KRAS pathway interacts with multiple downstream effectors, such as the RAF-MEK-ERK and PI3K-Akt-mTOR pathways, to regulate diverse cellular functions.^[44–46]^ Moreover, KRAS mutation is closely related to ferroptosis.^[47]^ Ferroptosis is induced in the KRAS-nutant colorectal cancer cells by inhibiting AMPK/AKT/mTOR signaling. Therefore, we speculate that 6 FPMMs modulate ferroptosis in BC through KRAS signaling. The specific mechanisms need more validation experiments.

It has been proposed that ferroptosis within the tumor microenvironment creates an advantageous environment for enhancing tumor survival and progression. This phenomenon arises from the infiltration and polarization of pro-tumorigenic immune cells, coupled with the compromised anti-tumor immune function.^[48]^ To further explore the functions of the identified 6 FPMM-related prognostic genes, we performed an immune infiltration analysis. The findings identified 5 immune cells (B cells, macrophages M1, T cells CD4, Tregs, and DCs) significantly related to 6 prognostic genes and risk scores. In the high-risk group, the infiltration levels of M1 macrophages, Tregs, and DCs were significantly increased, while B cells and CD4 + T cells were markedly decreased. This altered immune landscape may, to some extent, account for the poorer prognosis observed in the high-risk group. M1 macrophages have been shown to secrete factors that promote metastasis in epithelial-like BC.^[49]^ Moreover, macrophages play a crucial role in the ferroptosis-mediated promotion of the tumor immune microenvironment, and inhibiting ferroptosis may prevent macrophage polarization towards the M1 pro-inflammatory phenotype.^[50]^ As a typical immunosuppressive cell population, Tregs are significantly increased in the high-risk group and may suppress the activity of effector T cells, thereby dampening anti-tumor immunity. Studies have shown that Treg infiltration is associated with BC progression and adverse clinical outcomes.^[51]^ Similarly, although DCs are essential for antigen presentation, their function may be impaired or tolerogenic in the tumor microenvironment, leading to ineffective T cell activation.^[52]^ On the other hand, the reduction in B cells and CD4 + T cells may indicate a weakened adaptive immune response. GPX4 plays an essential role in protecting B cells from ferroptosis, and impaired GPX4 function can reduce B cell activity and survival.^[53]^ CD4 + T cells, which are critical for orchestrating immune responses, when decreased, may further compromise the body’s immune surveillance capability. Our findings suggest that in high-risk BC patients, ferroptosis-related processes may influence the composition and function of immune cells, thereby creating an immunosuppressive or dysfunctional tumor microenvironment that contributes to poor prognosis.

## 5. Conclusion

In conclusion, the present study elucidated the role of FPMMs in BC and developed a novel prognostic risk model based on 6 FPMMs. The construction and validation of the prognostic risk model demonstrated its potential clinical utility in predicting patient outcomes. Furthermore, expression and mutation analysis provided insights into the dysregulation of FPMM-related genes in BC and their association with patient prognosis. Despite the promising findings, further research is warranted to validate the clinical applicability of these findings, ultimately advancing personalized therapeutic strategies for BC patients.

## Author contributions

**Conceptualization:** Xue Han.

**Data curation:** Xue Han.

**Formal analysis:** Xue Han.

**Investigation:** Wurina Chen.

**Methodology:** Wurina Chen.

**Supervision:** Wurina Chen.

**Writing – original draft:** Xue Han, Wurina Chen.

**Writing – review & editing:** Wurina Chen.
